# A health economic evaluation of screening and treatment in patients with adolescent idiopathic scoliosis

**DOI:** 10.1186/s13013-014-0021-8

**Published:** 2014-12-06

**Authors:** Raphael D Adobor, Paal Joranger, Harald Steen, Ståle Navrud, Jens Ivar Brox

**Affiliations:** Section for Spine Surgery, Department of Orthopedic Surgery, Oslo University Hospital-Rikshospitalet, Pb 4950, Nydalen, N-0424 Oslo, Norway; Oslo and Akershus University College of Applied Sciences, P. O Box 4, St. Olavs plass, N-0130 Oslo, Norway; School of Economics and Business, Norwegian University of Life Sciences, P. O. Box 5003, N-1432 Ås, Norway; Biomechanic lab, Department of Orthopedic Surgery, Oslo University Hospital-Rikshospitalet, Pb 4950, Nydalen, N-0424 Oslo, Norway; Department of Physical Medicine and Rehabilitation, Oslo University Hospital and University of Oslo, Pb 4950, Nydalen, N-0424 Oslo, Norway

**Keywords:** Cost minimisation analysis, Scoliosis screening, Scoliosis treatment, Health related quality of life

## Abstract

**Summary of background data:**

Adolescent idiopathic scoliosis can progress and affect the health related quality of life of the patients. Research shows that screening is effective in early detection, which allows for bracing and reduced surgical rates, and may save costs, but is still controversial from a health economic perspective.

**Study design:**

Model based cost minimisation analysis using hospital’s costs, administrative data, and market prices to estimate costs in screening, bracing and surgical treatment. Uncertainty was characterised by deterministic and probabilistic sensitivity analyses. Time horizon was 6 years from first screening at 11 years of age.

**Objective:**

To compare estimated costs in screening and non-screening scenarios (reduced treatment rates of 90%, 80%, 70% of screening, and non-screening Norway 2012).

**Methods:**

Data was based on screening and treatment costs in primary health care and in hospital care settings. Participants were 4000, 12-year old children screened in Norway, 115190 children screened in Hong Kong and 112 children treated for scoliosis in Norway in 2012. We assumed equivalent outcome of health related quality of life, and compared only relative costs in screening and non-screening settings. Incremental cost was defined as positive when a non-screening scenario was more expensive relative to screening.

**Results:**

Screening per child was € 8.4 (95% CrI 6.6 to10.6), € 10350 (8690 to 12180) per patient braced, and € 45880 (39040 to 55400) per child operated. Incremental cost per child in non-screening scenario of 90% treatment rate was € 13.3 (1 to 27), increasing from € 1.3 (−8 to 11) to € 27.6 (14 to 44) as surgical rates relative to bracing increased from 40% to 80%. For the 80% treatment rate non-screening scenario, incremental cost was € 5.5 (−6 to 18) when screening all, and € 11.3 (2 to 22) when screening girls only. For the non-screening Norwegian scenario, incremental cost per child was € -0.1(−14 to 16). Bracing and surgery were the main cost drivers and contributed most to uncertainty.

**Conclusions:**

With the assumptions applied in the present study, screening is cost saving when performed in girls only, and when it leads to reduced treatment rates. Cost of surgery was dominating in non-screening whilst cost of bracing was dominating in screening. The economic gain of screening increases when it leads to higher rates of bracing and reduced surgical rates.

**Electronic supplementary material:**

The online version of this article (doi:10.1186/s13013-014-0021-8) contains supplementary material, which is available to authorized users.

## Introduction

Adolescent idiopathic scoliosis (AIS) is a complex three dimensional deformity of the spine, characterized by lateral curvature >10° and axial rotation, which affects 2-3% of otherwise healthy teenagers [1-3]. The deformity usually progresses with rapid growth of the spine and can affect health related quality of life of the patients [4]. Conventional treatment options are bracing and surgery [1-3]. Bracing is normally recommended for progressive curves of 20-40° in immature patients to prevent progression and reduce surgery, whilst surgery is considered for curves >45°-50° to stop progression and correct the deformity [1]. In patients with AIS, only a minority have progressive curves requiring treatment [5], and 90% of those treated are girls [6,7]. Treatment outcomes are usually measured by radiographic changes of the curves, but increasingly also by changes in health related quality of life. Early detection by screening allows for monitoring curve progression, and timely initiation of bracing. A recent randomised study found bracing to reduce curves which progress to the threshold of surgery [5].

Screening is controversial and practices vary worldwide [8-10]. Opponents cite mainly increased costs and lack of effectiveness of the programs. Some previous studies have supported whilst others have discouraged screening [11,12]. The United States Preventive Services Task Force neither supported nor opposed screening in 1993 [12,13], but recommended against routine screening in 2004 [14]. Discontinuation of screening programs has led to late detection and high rates of surgeries in various countries [15-17]. Currently, most international scoliosis and child health societies support and recommend screening [18]. The Scoliosis Research Society’s International task force recently reported even before the BRAIST study [5] was published, that screening was effective in technical, clinical, program, and treatment efficacy, but could not make a statement on cost effectiveness due to lack of studies evaluating costs and health economic analyses [19].

Reviews and long-term studies suggest that health related quality of life of patients treated with brace or surgery are not different [1,2,6]. The aim of the present study was therefore to perform a cost minimization analysis (CMA) comparing only costs in screening and non-screening settings, while assuming equal long term health related quality of life of patients whose scoliosis are detected through screening or without.

## Methods

We used a model approach to compare costs in screening with non-screening scenarios. The main mathematical equation on which the model was based is shown in Additional file 1. Input model parameters were collected from screening and hospital care. Screening in Norway was performed once in 12-year old children, and did not detect patients suitable for bracing [20]. We assumed similar epidemiology and natural history of AIS in Hong Kong and Norway, and used suitable data from a large population-based cohort longitudinal screening study by Lee et al. from Hong Kong in 2010 as model input for screening [21]. In this study, 115190 children were screened: 3158 received X-rays, 59 had out-patient visits for further assessment only, 264 were braced, 10 had surgery, and 29 had both brace and surgery (85% brace and 15% surgery). The percent treated in Hong Kong was thus 2.63 per 1000 children.

Screening is no longer performed in Norway. According to administrative data from the three scoliosis clinics in Norway, 122 adolescents were treated for scoliosis in 2012, of which 51(42%) were braced and 71(58%) had surgery, with about 10% of them having both brace and surgery. These 122 children, aged 11 to 17 is the number of patients out of the cohort of 63421 children who were the target group for scoliosis treatment in Norway for that year. Thus, the percent of children treated in Norway in 2012 was 1.92 per 1000 children.

Model input for the non-screening scenarios were based on Norwegian data when available. Otherwise, inputs were estimated from the Hong Kong data.

### Study perspective in relation to costs

We used a health sector budget perspective focusing on the costs related to orthopaedic treatment in hospital care [22], and in addition, we included costs for the society due to transportation and parents’ opportunity cost of time during treatment of their children.

#### Strategies being compared

Screening for scoliosis may lead to over-referrals to X-rays and outpatient evaluations, increased rates of bracing, but reduced surgical rates compared to settings when children are not screened [23,24]. In non-screening settings, many children are diagnosed late when they are matured, with curves not suitable for bracing [15-17,23]. We therefore assumed that reduced numbers of children are treated for scoliosis in non-screening settings and estimated reduced treatment rates of 90%, 80%, and 70%, respectively of those treated in screening by Lee et al. We compared costs in these reduced treatment rates to costs in the screening setting in Hong Kong. Treatment in this context includes the percentage of children who have X-rays for diagnosis, those treated with brace or surgery, and those who have further follow-ups. The estimated treatment rate of non-screening in Norway 2012 was 73% of that in Hong Kong. We also compared costs in non-screening scenario in Norway 2012 with the costs in the screening setting in Hong Kong. Since AIS is more prevalent in girls, and 90% of those treated for AIS are girls [5,6], we performed separate analyses in girls.

In all non-screening scenarios, we simulated different distribution rates of brace and surgery based on the available non-screening data from Norway (58% surgery and 42% brace), since this is the only available data on the distribution of brace and surgery in a non-screening setting. We used data from Hong Kong to estimate the frequency of X-ray examination and referrals since non-screening Norwegian data was not available (see Additional file 1). Based on this study, we estimated that about 15% of children required referrals to X-ray and to specialist’s examinations. In all non-screening scenarios, these rates were adjusted accordingly.

Incremental cost was defined as the cost of treatment in a non-screening scenario minus the cost of treatment and cost incurred in conducting the screening. A positive incremental cost therefore implies that screening is more cost saving compared to the non-screening scenario. How incremental cost changes by varying the ratio of bracing to surgery was estimated for all the non-screening scenarios. The probability of the incremental cost being positive was estimated for all cases.

#### Time horizon for cost estimations, discount rate

The time horizon for estimating costs was six years from the first screening at 11 years of age. We assumed two screenings per child, based on the recommendations of the Scoliosis Research Society [18] at the age of 11 and 13 years, and anticipated that 60% of the scoliosis cases were detected at the first screening and the rest at the second. We based our assumption on the knowledge of age and gender- specific prevalence of scoliosis, as well as the length of time between detection and treatment. Since screening tests are not fully accurate, it has also been suggested that scoliosis screening programs should be planned as a continuous process and not just a once and for all project as there is a possibility of missing out on some cases if screening is performed only once. For the non-screening scenarios we also assumed a dispersion of the expected cost (bracing and surgery) of 10%, 15%, 20%, 20%, 15% 10%, and 10% for each age group from 11 to 17 respectively. The literature is scarce with regards to the true dispersion of expected costs in scoliosis treatment, but shows a peak of treatment around 13–14 years of age. We therefore assumed 25% expected costs before, and 35% after the peak years [2,5,6,25]. When aggregating costs over time, we used an annual social discount rate of 4% (as recommended by the Norwegian Directorate of Health [26]) to calculate the present value of costs. The social discount rate is an interest rate used to bring future value into the present when considering the time value of money [22].

### Estimating costs and resources

We used hospital’s costs and administrative data, and market prices to estimate the cost of screening, bracing and surgery.

#### Screening

Screening was performed once in 4000 twelve year old children as part of a vaccine and physical examination program from autumn 2006 to spring 2007 [20]. Community nurses and physical therapists performed the screening. All activities directly involved in the screening and follow-up of patients were identified, measured, and costs estimated (Table 1).

**Table 1:** **Resource unit used, cost (€) per unit, number of units and the uncertainty interval used for the cost estimation in the probabilistic sensitivity analysis (PSA)**

**No.**	**Variables**	**Unit cost (€)**	**Range (±%), cost**	**Units**	**Range (±%), units**
	**Screening**				
1	Examiners (minutes)	47	20	9	20
2	Materials and supplies	0.03	20		
3	Scoliometer	1.4	20		
	For confirmation of scoliosis				
4	Transportation to X-ray exam	22	50		
5	Radiographs	63	30		
	For confirmed scoliosis >20°				
6	Transport to specialist evaluation	182	50		
7	Specialist evaluation	62	30		
8	Radiographs	128	30		
	**Brace treatment**				
9	Boston brace	3020	20	1.5	30
10	Reimbursement for wear and tear of clothes and linen/year	725	20	2	20
11	Hospital hotel, days (child and 1 parent)	212	30	3	30
12	Out-patient consultations	62	30	4	20
13	Physical therapy	55	30	1	20
14	Radiographs	128	30	4	20
15	Time used by one parent (days)	289	30	4	30
16	Transportation	137	50	4	50
	**Surgery**				
17	Implants/utilities (per operation)	9390	20		
18	Out patients consultations	62	30	4.5	10
19	Surgeons (hours)*	118	30	6	20
20	Anesthesiologists (hours)	118	30	5	20
21	Anesthesiologist nurse (hours)	71	30	5	20
22	Scrub nurses (hours)*	71	30	10	20
23	Intensive care (days)	4190	30	1	
24	Postoperative care unit (per day)	1872	30	2	25
25	Regular ward (days)	1541	30	8	25
26	Physical therapy	55	30	10	20
27	Radiology examination	160	30	6	20
28	Time used by one parent (days)	289	30	15	30
29	Taxi from home to school after treatment (days)	63	50	10	50
30	Transportation (days)	104	50	6	30
31	Transportation home after surgery	508	50		

*Two surgeons and two scrub nurses were involved in each surgery.

All items in each category of interventions were identified, measured, and costs estimated. Percentage of uncertainty was estimated for each item. The percentages of the uncertainty of the PSA’s are also given.

#### Bracing and surgery

We estimated the costs of bracing and surgery based on data from hospital records. For bracing, we estimated the costs of the brace equipment, transportation, radiographic and clinical examinations during the period of brace wear, 3 days hospital hotel services for the child and one parent during brace fitting. Additionally, the costs of reimbursements for wear and tear of clothing and beddings from the National Insurance Scheme were included. For surgery, we estimated the costs of implants, salaries of the staff at the theatre, intensive care, intermediate postoperative care, regular ward costs, and costs of re-operations (Table 1).

Surgery was usually performed using either a hybrid construct with an average of 5 pedicle screws, 8 hooks, and 5 to 6 sublaminar wires or an all pedicle-screw construct using 15 to 17 pedicle screws. Two surgeons usually performed the surgery using an estimated average time of 180 minutes. One anesthesiologist, one anesthesiology nurse and two scrub nurses assisted them working on average for 300 minutes. After surgery, patients stayed in hospital for an average of 10 days. No braces were used postoperatively. During the first postoperative year, patients had two follow-up consultations. In addition, costs of radiological examinations, outpatient visits for follow-ups, transportation, and costs of complications and re-operations during the first year were measured.

With the public universal healthcare system in Norway, there are no hospital fees for parents when children are braced or surgically treated. Cost per hour for different health professionals was estimated by adding social costs of employment (pension, insurance, sick-leave, and training) and overhead to the salary (inclusive income tax). The salary and social costs for hospital staff were estimated using the mean salary at the Oslo University Hospital and the estimates of the overhead costs were based on data from the Norwegian Central Bureau of Statistics [27]. Salary and social costs of public health nurses were based on data from the Norwegian Nurses organization, and local community administrations.

#### Currency, price date and conversion

All prices and costs were converted from 2006 to 2012 NOK (Norwegian kroner) by using an inflation rate of 3.21% per year based on the yearly rate of change of one unit value within the Diagnosis-Related Group (DRG) System in Norway. The exchange rate used was 8 NOK =1 € (Euro).

### Statistical analysis

**Figure 1:**
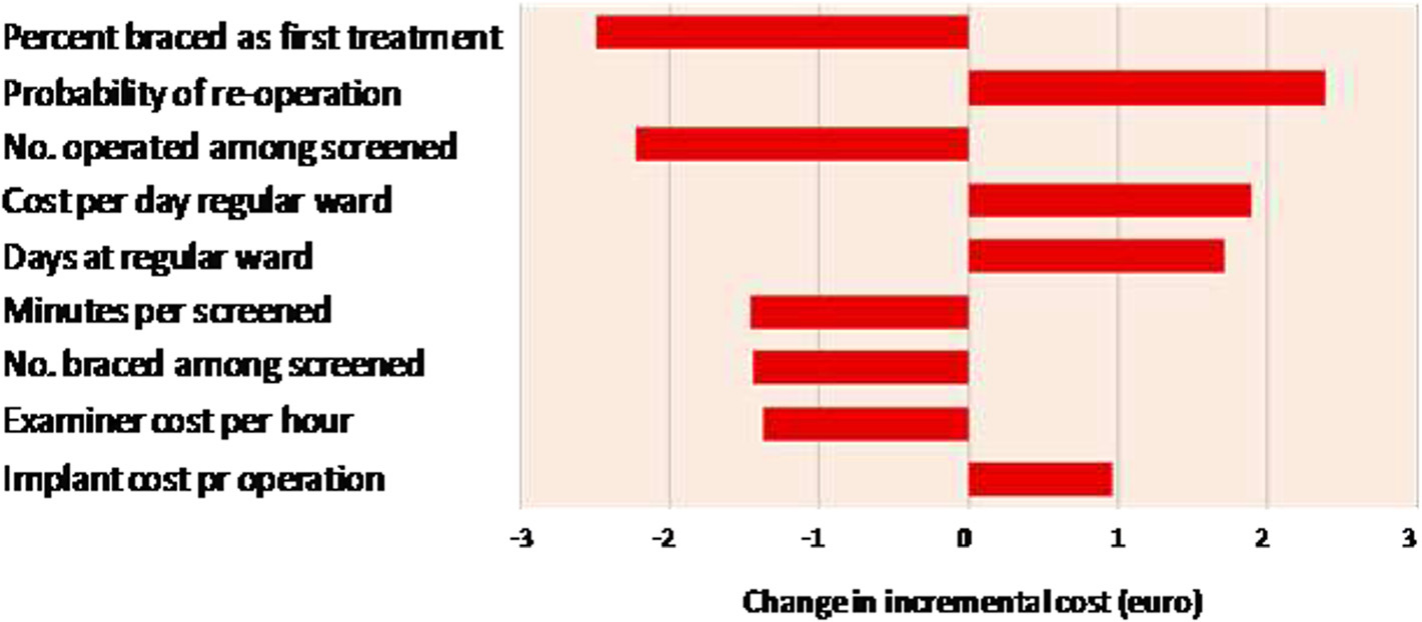
**Tornado diagram (sensitivity analysis) for comparing the 80% treatment rate of Lee et al. non-screening scenario to screening.**

Values are given as numbers, percentages, means and mean differences. Results are presented with a 95% credibility interval (CrI), which show the 2.5th and 97.5th percentile of the outcome distribution. The uncertainty of input variables was assessed by one-way and multi-way sensitivity analyses. Parametric uncertainty was analyzed by probabilistic sensitivity analysis (PSA), where all uncertainties in the relevant parameters were accounted for simultaneously [22,28]. The PSA was used to analyse the distribution of incremental cost estimations in all scenarios (100000 interactions) and to estimate the CrI for total incremental costs, which forms the basis for the Tornado diagram in Figure 1. In the PSA, we used gamma distributions for estimation of unit costs, beta distributions for the number of hours used and their probabilities. Poisson distributions were used for the number of children treated.

The screening study was approved by the Regional Ethical Committee for Medical Research in Norway.

## Results

### Cost estimations

For all the relevant scenarios, the total estimated costs were € 8.4 (95% CrI 6.6 to 10.6) per child screened, € 10350 (8690 to 12180) per patient braced, and € 45880 (38040 to 55400) per surgery (re-operations included). The average time used to screen a child was 9 minutes (Table 1).

#### Incremental costs and outcomes

The incremental cost per child in a non-screening scenario of 90% treatment rate compared with screening was € 13.3 (1 to 27). The probability of the incremental cost being positive was 99%. In the 80% treatment rate non-screening scenario, incremental cost was € 5.5 (−6 to 18) with the probability of the incremental cost being positive was 82%. When comparing non-screening scenarios to screening for girls only: the incremental cost was € 11.3 (2 to 22) for the 80% treatment rate scenario and € 4.3 (−4 to 14) for the 70% treatment rate scenario. The probability of the incremental cost being positive was 99% and 82%, respectively. The incremental cost per child in the non-screening Norwegian scenario compared with screening was € 0.1 (−14 to 16), and the probability of the costs being positive was 50% (Table 2).

**Table 2:** **Cost (€) per alternative (screening boys and girls combined vs girls only) and incremental cost relative to screening in four non-screening scenarios with a 95% Credibility Interval (CrI)**

	**Screening boys and girls**			**Screening girls only**		
	**Cost per child**	**Incremental cost per child**	**Probability incremental cost >0**	**Cost per child**	**Incremental cost per child**	**Probability incremental cost >0**
**Screening**	57.0 (49 to 66)	-		50.6 (44 to 58)	-	
**Non-screening Norway**	57.1 (44 to 73)	0.1 (−14 to 16)	50%	57.1 (44 to 73)	6.5 (6 to 21)	84%
**Non-screening 90% treatment rate of Lee et al.**	70.3 (59 to 84)	13.3 (1 to 27)	99%	70.3 (59 to 85)	18.4 (8 to 30)	>99%
**Non-screening 80% treatment rate of Lee et al.**	62.5 (52 to 75)	5.5 (−6 to 18)	82%	62.5 (52 to 75)	11.3 (2 to 22)	99%
**Non-screening 70% treatment rate of Lee et al.**	54.7 (46 to 66)	−2.3 (−13 to 9)	33%	54.7 (46 to 66)	4.3 (−4 to 14)	82%

The incremental cost was highest in the 90% treatment rate non-screening scenario with probability of being > 0 close to 100%. Incremental cost in non-screening Norway 2012 is close to the 70% treatment rate scenario. Incremental costs were higher in all non-screening scenarios when comparing screening of girls only than when comparing to screening of both boys and girls. The probabilities of incremental costs being >0 are also higher when comparing non-screening scenarios to screening of girls only than for both boys and girls combined.

Comparing the undiscounted cost per child in the 80% treatment rate non-screening scenario, to screening, the cost of bracing per child of € 26.0 (21 to 33) was dominating in the screening scenario, whilst the cost of surgery per child of € 60.2 (48 to 75) was dominating in the non-screened scenario.

Incremental cost in the non-screening 90% treatment rate scenario varied from € -6.3 (−13 to 3) to € 27.6 (14-42) as the percentage of surgery increased from 30% to 80%. For the 80% treatment rate scenario with 30% surgery, and 70% bracing, incremental cost was € -11.0 (−19 to −3) favouring non-screening. With 80% surgery, and 20% bracing, incremental cost was € 18.2 (6 to 33) favouring screening (Table 3).

**Table 3:** **Incremental costs in non-screening scenarios compared with screening**

		**Ratios of brace/surgery in non-screening scenarios**					
		**20/80**	**30/70**	**40/60**	**50/50**	**60/40**	**70/30**
Treatment rates in non-screening scenarios compared to screening	100%	**37.0** (22 to 55)	**29.7** (16 to 45)	**22.4** (10 to 36)	**15.1** (4 to 27)	**7.8** (−2 to 18)	**-0.5** (−8 to 9)
	90%	**27.6** (14 to 44)	**21.0** (9 to 35)	**14.5** (3 to 27)	**7.9** (−2 to 19)	**1.3** (−8 to 11)	**- 5.3** (−13 to 3)
	80%	**18.2** (6 to 33)	**12.4** (1 to 25)	**6.5** (−4 to 18)	**0.7** (−9 to 11)	**- 5.2** (−14 to 4)	**- 11.0** (−19 to −3)
	70%	**8.8** (−3 to 22)	**3.7** (−7 to 15)	**- 1.4** (−11 to 9)	**- 6.5** (−15 to 3)	**- 11.6** (−20 to −3)	**- 16.8** (−24 to −9)
	60%	**- 0.6** (−11 to 11)	**- 5.0** (−15 to 5)	**- 9.3** (−18 to 0)	**- 13.7** (−22 to −5)	**- 18.0** (−26 to −10)	**- 22.5** (−30 to −15)

Mean 95% Crl are given for non-screening scenarios with treatment rates from 60% to 100% combined with different ratios of bracing to surgery from 20/80 to 70/30.

Non-screening is more expensive with higher treatment rates and higher surgical rates compared with screening. Non-screening is less expensive with lower treatment rates and higher bracing rates compared to screening.

#### Characterizing uncertainty

**Figure 2:**
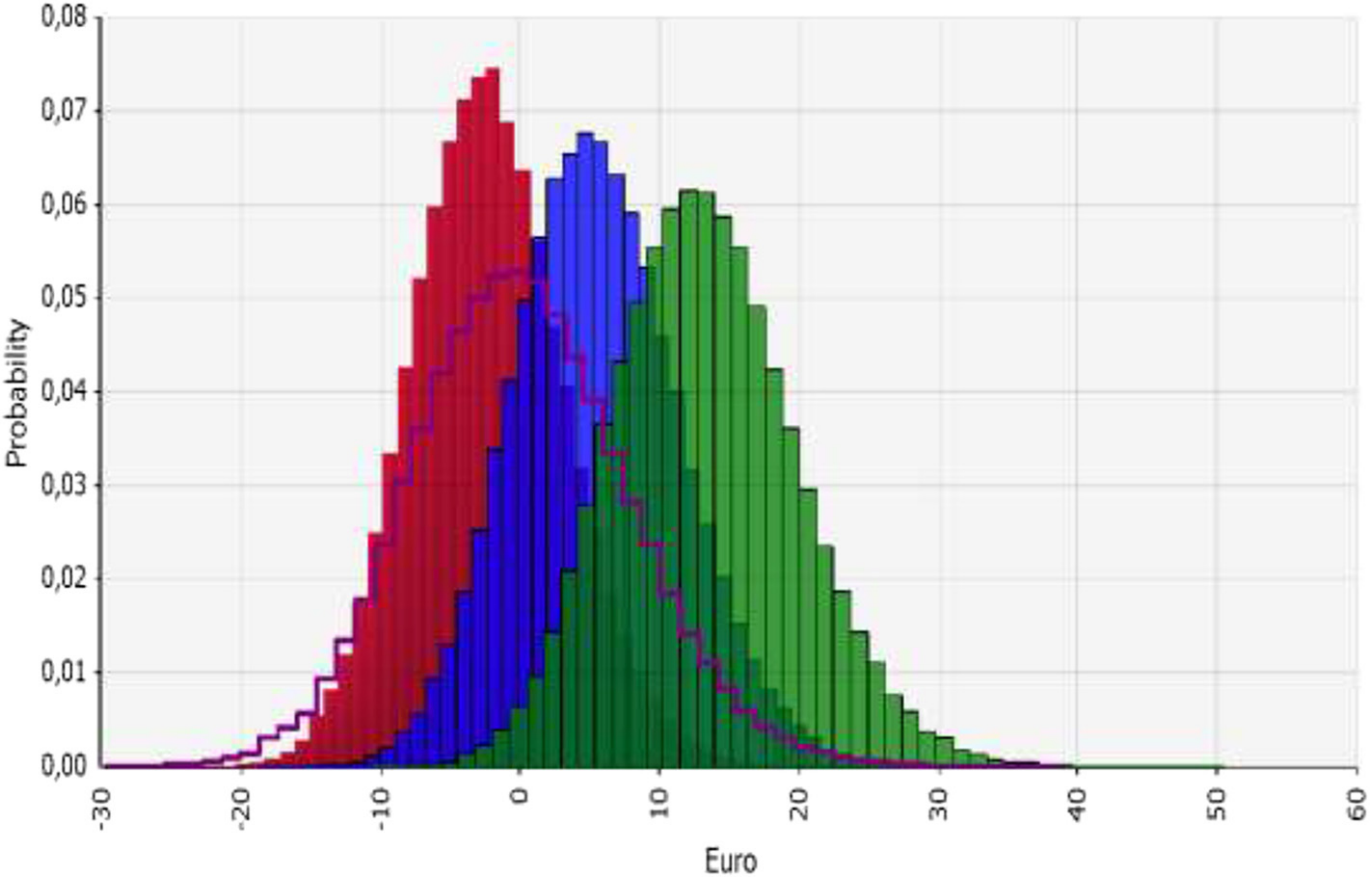
**Incremental cost estimations in four non-screening scenarios compared to screening both boys and girls.** Incremental costs increase from left to right looking at the top of the curves. Incremental cost was lowest in non- screening 70% treatment rate of Lee et al (red), followed by Norway (purple) 80% treatment rate of Lee et al (blue), and 90% treatment rate of Lee et al (green). Incremental costs were highest with higher treatment rate non-screening scenarios and lower in low treatment rate non- screening scenarios compared to screening of both boys and girls. The areas under the curves to the right of zero equals the probabilities of incremental costs being >0.

**Figure 3:**
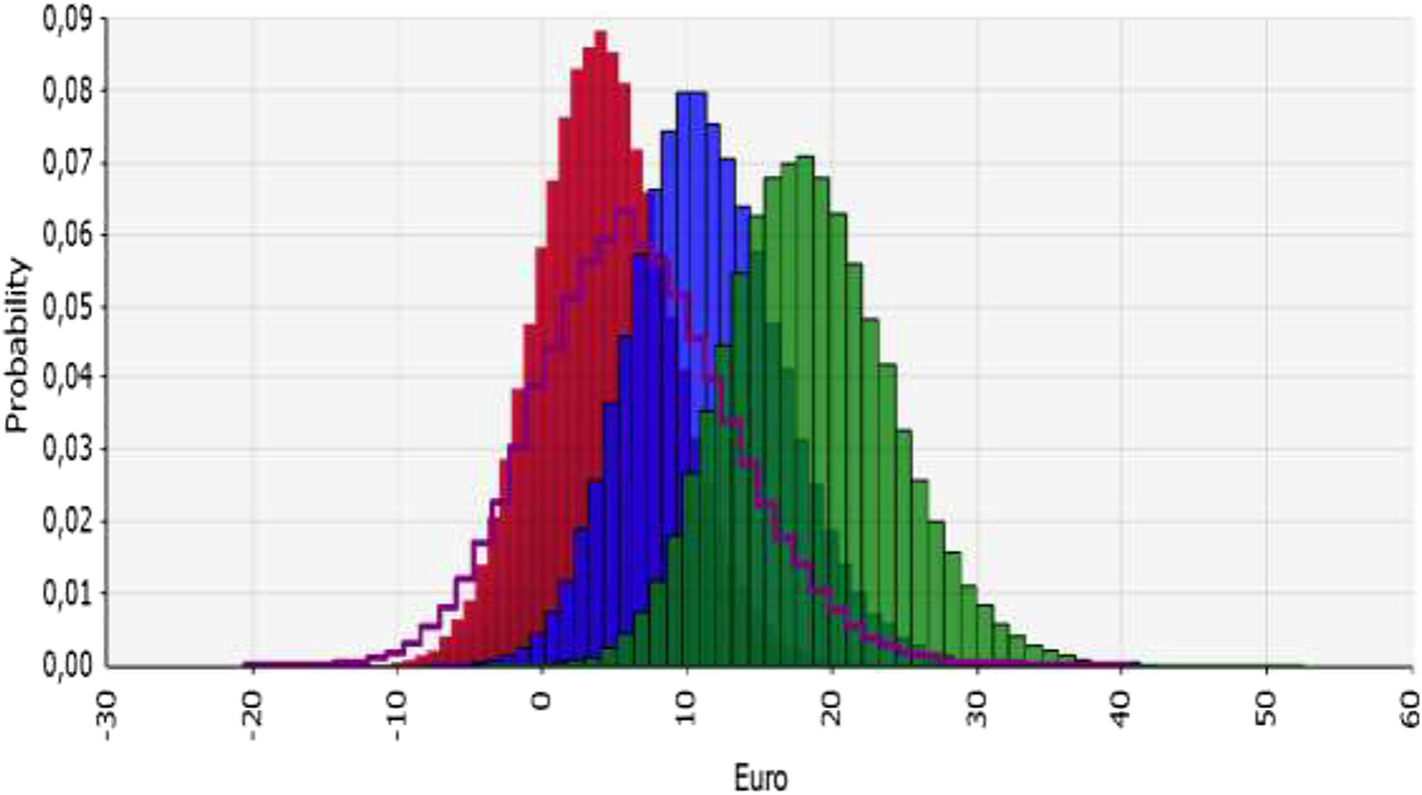
**Incremental cost estimations in four non-screening scenarios compared to screening of girls only.** Incremental costs increase from left to right looking at the top of the curves. Incremental cost was lowest (cost saving) in the 70% treatment rate of Lee et al (red), followed by non-screening Norway (purple), the 80% treatment rate of Lee et. al non screening scenario (blue), and the 90% treatment rate of Lee et al. non-screening scenario (green) compared to screening girls only. The areas under the curves to the right of zero equals the probabilities of incremental costs >0 which are considerably higher when comparing non-screening scenarios to screening of girls only than when comparing non-screening scenarios to screening of both boys and girls (Figure 2).

The expected incremental cost estimates are shown in Figure 2. In the 90% treatment rate non-screening scenario, the probability of a positive incremental cost was close to 100%. Results comparing non-screening scenarios to screening in girls are shown in Figure 3. Uncertainty is also illustrated in the tornado diagram for the non-screening scenario of 80% treatment rate. The most important contributor to uncertainty was the percent braced, followed by the probability of being re-operated (Figure 1).

## Discussion

Scoliosis screening programs are considered to be beneficial from a clinical point of view [19], but are criticized for high costs due to high referral and treatment rates [8,11,13]. In the present study we used data from a large longitudinal screening study, and detailed costing of all activities in performing the analyses. Results suggest that screening is cost saving, unless both treatment rates and surgical rates are very low in comparative non-screening scenarios. In agreement with previously published studies reporting that discontinuation of screening has led to late detection and high rates of surgery [15-17], the model applied in the present study indicates that costs increase in non-screening scenarios with high rates of surgery and lower rates of bracing.

The effectiveness of a screening program thus depends on the costs involved and the number of cases detected early that result in bracing and less surgery compared to a non-screening setting. In a recent clinical trial, bracing reduced the number of children with curve progression to the threshold of surgery [5].

The results of the present study show that, screening has a large potential of cost saving if only girls are screened. Selective screening of girls is most cost saving because they constitute about 90% of those treated for scoliosis. In Table 2, we showed that there is a high probability of cost saving when only girls are screened compared to non-screening scenarios with treatment rates widely ranging from 70% to 100% of those of screening.

Table 3 shows that in the extreme non-screening scenario where treatment rates are approaching those of screening, screening both boys and girls was not cost saving. Likewise in the extreme non-screening scenario where treatment rates were very low approaching 60% of those treated in screening, non-screening becomes cost saving. However, these scenarios are the least likely to occur. In the non-screening scenarios where treatment levels are 90-100% of those in screening, patients are probably younger at detection, and likely to be recommended bracing according to guidelines and the results of the recent RCT study on bracing [5]. This implies that the ratio of bracing/surgery is likely to be >1 and bracing will be the dominating treatment option. On the contrary, when treatment levels in non-screening scenarios are in the 60% to 70% range of that of screening, patients are likely to be older and curves too large and not suitable for bracing [15], and surgery is most likely to be the dominating treatment option (i.e. ratio of brace to surgery likely to be <1).

In the Hong Kong study, about 15% of those detected by screening ended up having surgery compared to about 60% in non-screening Norway. Obviously, screening is not cost saving if the number treated in non-screening approximates that with screening and the surgical rate is 15%. However, this scenario is very unlikely to occur and was therefore not included in our analyses.

An interesting finding according to Table 3 is that screening both boys and girls tends to increase costs if the distribution of brace/surgery is 70/30 or 60/40 in a non-screening scenario. This scenario is also unlikely to occur. According to a previous Norwegian study non-screening scenarios of 30/70 or 40/60 are more likely to occur [15].

Our findings are in agreement with a review [29] on cost effectiveness of screening that found screening to be cost effective in one study [30], and recommended screening only for high-risk groups such as girls at twelve years of age in order to reduce over-referrals and over-treatment. However, the most recent review was not able to conclude whether screening was cost effective or not [31]. None of the studies cited in these reviews, however, applied recommended health economic evaluation principles [32].

Simulations in the present study suggest that the economic gain of screening increases when screening leads to higher rates of bracing and reduced rates of surgery. In a previous study, we reported higher rates of bracing and reduced surgical rates during a period of screening compared to a period without [15]. Similar findings have been reported from the Netherlands, Sweden and USA [23,24,33]. Bracing has been shown to reduce progression of curves to the threshold of surgery. In the recently published RCT study on bracing, the success rate was >70% and about 90% in those with high compliance [5]. Similar results were observed at long-term in a large Norwegian cohort study [6]. The current evidence of efficacy of bracing in the short term and good results at long-term indicates that patients with AIS should be detected early to allow for bracing. In addition, bracing avoids the complications of surgery, keeps the spine mobile, and might have positive long term effects. These benefits should be considered when interpreting the results of the present study. There has however been a lack of enthusiasm for bracing in the past amongst care providers. This is presumably due to the absence of high level of evidence of efficacy on bracing, and concerns of negative psychological impact on the patients. The results from the recent RCT study [5] on bracing do not however support this view.

With the assumptions made in the current study, screening of both boys and girls would neither have increased nor decreased costs compared to the treatment of AIS in Norway in 2012 where the estimated treatment rate was 73% compared to screening in Hong Kong, and 58% had surgery. However, selective screening of girls only would have been cost saving in Norway; as shown in Table 2 above.

Studies in the past have reported varying costs of scoliosis screening, and costs of bringing cases detected on screening to treatment, depending on how costs are measured [30,34-39]. The cost of screening in the current study is comparable to similar programs in Europe where total costs were included [34-36]. The estimated cost was based on two screenings per child, and community nurses performed the screening in conjunction with a vaccine program. Transportation costs and salaries of health professionals would have increased if screening had been performed in a different and isolated setting and not by community nurses. The estimated costs of bracing and surgery are comparable to those reported in the literature [40]. Many factors may influence the validity of our cost estimations. Treatment costs are likely to be underestimated in our study as bone grafts and intra-operative neuromonitoring were not used during surgeries, as compared with a study from the USA [40]. Our study perspective was limited to costs related only to expenses in an orthopedic department. We did not include costs related to primary health care, paramedics and alternative costs in relation to referred patients. In addition, we did not systematically register costs of patients’ out- of- pocket expenses like transportation in relation to adjuvant treatment for scoliosis. Though physical therapy and counseling are not routinely offered to AIS patients in Norway, it is estimated that 1/3 of the patients use physiotherapy whilst under brace treatment or postoperatively [6,41].

Several input parameters contribute to uncertainties in our analysis. The cost of regular wards in surgical treatment was difficult to estimate accurately despite considerable effort. AIS patients undergoing surgical treatment require increased nursing resources compared to caring for ordinary pediatric patients at the orthopedic ward. The main analyses may also underestimate the cost of surgery.

The probabilities of positive incremental costs varied widely in the current study. There was however higher certainty in the incremental cost estimates when comparing non-screening scenarios to screening of girls only, as opposed to boys and girls combined. More research is warranted in order to reduce the uncertainties in future health economic evaluations of scoliosis treatment.

### Limitations and strengths

Ideally, randomised studies or controlled prospective studies are needed to compare outcome in scoliosis treatment detected through screening or otherwise. However since the prevalence of scoliosis is low, it is difficult to include an adequate study sample even within a large country or internationally. Clinical trials including utility comparisons of bracing and surgery in both short and long terms are lacking. Utility scores may differ in shorter periods during treatment, for example by wearing a rigid brace, or postoperatively.

We assumed similar prevalence and natural history of AIS in Hong Kong and Norway in performing the analysis. Studies, however, show regional variations in the prevalence of AIS, like higher prevalence in girls, but not boys in higher latitudes than in lower latitudes [42]. However, those differences could be linked to environmental factors such as the difference in the onset of menses in different geographic locations [43], and different cultures and not related to genetics. It is also likely that mechanisms of referral may be very different in the two settings, and in various countries, due to healthcare systems structures and barriers to access. The presentation of AIS has also been reported to be linked to socioeconomic status and race [44]. A recent study however found equal prevalence of AIS in 12- year old children in Malaysia and Norway [20,45].

The main strength of the present work is the application of current recommended standards for reporting health economic evaluations in conducting the study [32]. This gives more transparency and complete reporting of methods and findings which will facilitate interpretation and comparison of similar studies. We also used data from the largest reported longitudinal study of screening cohorts [21]. Analyses were performed to assess the uncertainties. The percentage detected for bracing, costs of surgery, and re-operations were the major contributors to uncertainty. More accurate estimates of these factors could improve the reliability and applicability of future analyses.

### Generalisability

The model approach used in the current study could be employed worldwide with local cost estimate variations. Our results provide the missing economic evidence for health policy makers and healthcare providers to consider reintroduction of scoliosis screening.

In providing health services, policy makers are concerned about costs in view of limited healthcare resources, whereas patients and their families value the best treatment option available independent of costs. At present, there is a gap in the knowledge of the patient’s preference in choosing treatment options. In a recently published trial, bracing was preferred to observation by patients and their families leading to the interruption of the trial and subsequently continued as a preference study [5].

## Conclusions

Early detection through screening leading to bracing and fewer surgeries may save costs. Selective screening of high-risk groups like girls should probably be preferred. Screening is not likely to increase costs unless both treatment and surgical rates are very low in comparable settings where screening is not performed.

### Consent

Written informed consent was obtained from all patients for the publication of this report and any accompanying images.

## Additional file

Additional file 1:
**The mathematical model.**
