# Supplementary material for: A health economic evaluation of screening and treatment in patients with adolescent idiopathic scoliosis
Source: Scoliosis. 2014 Dec 6;9:21. doi: 10.1186/s13013-014-0021-8 (PMC4298059; doi:10.1186/s13013-014-0021-8)
Supplement: Additional file 1: — The mathematical model. [file 13013_2014_21_MOESM1_ESM.pdf]

Additional file 1: The mathematical model

A.1: Introduction

In this supplementary data, we show the core equation on which the simulation model was based. We begun by presenting the equations for estimating the cost of the different interventions: screening, diagnosis of scoliosis, confirming scoliosis > 20º, brace treatment and surgery. Then we estimated the fraction of children receiving each category of interventions in the various scenarios. In the end we merged the estimated costs and the estimated fractions to estimate the cost pr child for each category of intervention and for the different scenarios.

The methodology used in the cost-minimizing analysis and discounting are presented in the main text of the manuscript and based on general literature on health economic evaluation like Drummond et al[1] or Hunink et al[2]. Methods for performing decision models probabilistic are based on Briggs et al.[3] The simulation model was built in Microsoft Excel. For the probabilistic sensitivity analysis we used the software @risk which is a part of the Decision Tools Suite software. The software @risk works is an extension to Excel.

A2: Estimation of cost of screening, brace treatment and surgery – all scenarios

Estimating the cost of the school screening per examination:

$C_s = (u_1 \bullet uc_1) + m + s$

u_1 = Number of minutes (units) used pr child pr examination (se row 1 in Table 1).

uc_1 = Cost pr minute (unit cost) used pr examination (se row 1 in Table 1).

m = Cost of materials and supplies per examination.

s = Cost of scoliometer pr examination.

Estimating the cost of diagnosing one child for scoliosis:

$C_{con} = t_{con} + rad_{con}$

t_{con} = Cost of transportation to/from X-ray exam (se row 4 in Table 1).

rad_{con} = Cost of radiographs (se row 5 in Table 1).

Estimating the cost per confirmation of scoliosis > 20º:

$C_{con>20} = t_{con>20} + q_{con>20} + rad_{con>20}$

t_{con} = Transport to/from specialist evaluation (se row 6 in Table 1).

$q_{con>20}$ = Specialist evaluation (se row 7 in Table 1).

$rad_{con>20}$ = Radiographs (se row 8 in Table 1).

Estimating the cost of brace per treatment:

$C_b = \Sigma(u_j \bullet uc_j)$

Where j = 9 to 16 in table 1. For example for j = 11 are $u_{11} \bullet uc_{11}$ equal to 3 hospital hotel days multiplied with €212 per day in hospital hotel, and likewise for the other cost components of brace treatment.

Estimating the cost per operation:

$C_{su} = im + t + \Sigma(h_i \cdot hc_i) + \Sigma(u_j \cdot uc_j)$

im = Utilities/implants cost per operation

t = Cost for transportation home after surgery.

h_i = Hour used of health personnel in category i.

hc_i = Cost pr hour pr person of health personnel in category i.

u_j = Number of units used of category j.

uc_j = Cost pr unit of category j.

Where i = 18 to 21 in Table 1, and j = 22 to 30 in Table 1.

For each child receiving an operation, 15% were assumed to be re-operated. So, for per child operated, the cost will be 100% + 15% of the costs estimated by the equation above.

A3: Estimating the fraction of children receiving each category of interventions

A3.1The screened group

The fraction of the screened children receiving different categories of interventions is entirely based on the Hong Kong study:

$Fsc_j = TrHK_j / ChHK$

Fsc_j = The fraction of children in the screening group receiving intervention category j.

$TrHK_j$ = The number of children in the Hong Kong study receiving intervention category j.

ChHK = The number of children participating in the Hong Kong study.

Here, j = 31 to 34, were 31 means diagnosing scoliosis, 32 means confirming scoliosis > 20º, 33 means brace treatment and 34 means surgery.

A3.2The non-screening group

A3.2.1Non-screening scenario Norway

The fraction of children receiving surgery or brace treatment:

$FnscN_j = TrN_j/ChN$

$FnscN_j$ = The fraction of children receiving intervention category j.

TrN_j = The number of children 2012 in Norway receiving intervention category j.

Here, j = 33 and 34, were 33 means brace treatment and 34 means surgery. The number of surgery cases is the number of children receiving surgery only and the number of children receiving surgery after being braced.

ChN = The number of children in Norway in the age cohort of year 2012.

To estimate the number of children 2012 in Norway receiving surgery (TrN_{34}) we took the number of children receiving surgery as the first treatment option and added the 10% of the children receiving bracing as first treatment option because these children receive in addition surgery later on.

The fraction of the non-screened children confirmed for scoliosis or scoliosis > 20º:

$FnscN_j$ = $(((TrHK_j / ChHK) \bullet ChN) \bullet Fr\text{-}conf) / ChN)$ = $((TrHK_j / ChHK) \bullet Fr\text{-}conf)$

Fr-conf = The fraction of the screened children confirmed for scoliosis or scoliosis > 20º, who also would be confirmed for scoliosis or scoliosis > 20º if the same group when not screened. Fr-conf was assumed to be 0.15. The treatment rate for the Norwegian scenario was 73%. To change this according to the scenarios with different treatment rates, we adjusted the $FnscN_j$ to fit for the 80% scenario by multiplying $FnscN_j$ with 0.8, and for 70% scenario by multiplying with 0.7.

Here, j = 31 and 32, were 31 means confirmed for scoliosis and 32 means confirmed for scoliosis > 20º.

A3.2.2: Non-screening scenario 70%, 80% and 90%

We illustrate by using the 80% non-screening scenario. The same types of equations were used for the 70% and 90% scenarios.

The fraction of the children in a year cohort (or the chance pr child) receiving surgery or brace treatment for the 80% non-screening scenarios:

$Fnsc80_j = Tr80_j/ChN$

$Fnsc80_j$ = The fraction of children receiving category j treatment for the 80% non-screening scenario.

$Tr80_j$ = The number of children receiving category j of treatment in the 80% non-screening scenario.

Here, j = 33 and 34, were 33 means brace treatment and 34 means surgery.

In the 80% non-screening scenario, number receiving brace treatment and surgery, respectively:

$Tr80_{33} = ((TrN_{33}/(TrN_{33} + TrN_{34})) * TrNifHK) * 0{,}8$

$Tr80_{34} = ((TrN_{34}/(TrN_{33} + TrN_{34})) * TrNifHK) * 0{,}8$

$Tr80_{33}$ = The number receiving brace treatment for the 80% non-screening scenario.

$Tr80_{34}$ = The number receiving surgery for the 80% non-screening scenario. The number of surgical cases is the number of children receiving surgery only and the number of children receiving surgery after being braced.

TrNifHK = Total number treated with brace or surgery in Norway if the group was screened and treated as in the Hong Kong children. This parameter help us linking the fraction of treated when non-screened to the fraction of treated if screened – treatment among the non-screened is here 80% of the treatment among the screened.

$TrNifHK = \Sigma ((TrHK_j / ChHK) * ChN)$

Here, j = 33 and 34, were 33 means brace treatment and 34 means surgery.

Note that, when we use the notion "treatment rate" in the main text, we do not "double-count" the cases of surgery. Instead we refer to the rate of children treated by brace or surgery, where those who are both receiving braced and surgery are included among the braced.

A4: Estimating the cost pr child

A4.1: The screened group

Here we estimate the cost pr child in a cohort (defined as the selected one year cohort) for the different interventions.

$CChS_{sc} = 1 * C_s$

$CChS_{con} = Fsc_{31} * C_{con}$

$CChS_{con>20} = Fsc_{32} * C_{con>20}$

$CChS_b = Fsc_{33} * C_b$

$CChS_{su} = Fsc_{34} * C_{su}$

$CChS_{sc}$ = Cost of school screening pr child screened.

$CChS_{con}$ = Cost of confirming scoliosis pr child screened.

$CChS_{con>20}$ = Cost of confirming scoliosis > 20º pr child screened.

$CChS_b$ = Cost of bracing pr child screened.

$CChS_{su}$ = Cost of surgery pr child screened.

A4.2:The non-screening group

Here we use the 80% scenario as an example.

$CChN\text{-}S_{con} = FnscN_{31} * C_{con} * 0.8$

$CChN\text{-}S_{con>20} = FnscN_{32} * C_{con>20} * 0.8$

$CChN\text{-}S_b = Fnsc80_{33} * C_b$

$CChN\text{-}S_{su} = Fnsc80_{34} * C_{su}$

$CChN\text{-}S_{con}$ = Cost of confirming scoliosis pr child not screened.

$CChN\text{-}S_{con>20}$ = Cost of confirming scoliosis > 20º pr child not screened.

$CChN\text{-}S_b$ = Cost of bracing pr child not screened.

$CChN\text{-}S_{su}$ = Cost of surgery pr child screened.

These cost pr child pr intervention was dispersed over a 6 year period as described in the main text of the manuscript. The *incremental cost* was estimated by subtracting the total discounted cost pr non-screened child from the total discounted cost pr screened child.

Reference List

1. Drummond MF, Sculpher MJ, Torrance GW, O'Brien BJ, Stoddart GL: *Methods for Economic Evaluation of health Care Programmes*, 3 edn. Oxford Medical Publications; 2005.

2. Hunink M GPSJWJPJEAea: *Decision making in health and medicine - integrating evidence and values.* Cambridge University Press:New York; 2001.

3. Briggs AH, Claxton K SM: *Decision modelling for health economic evaluation*. Oxford: Oxford University Press; 2006.
